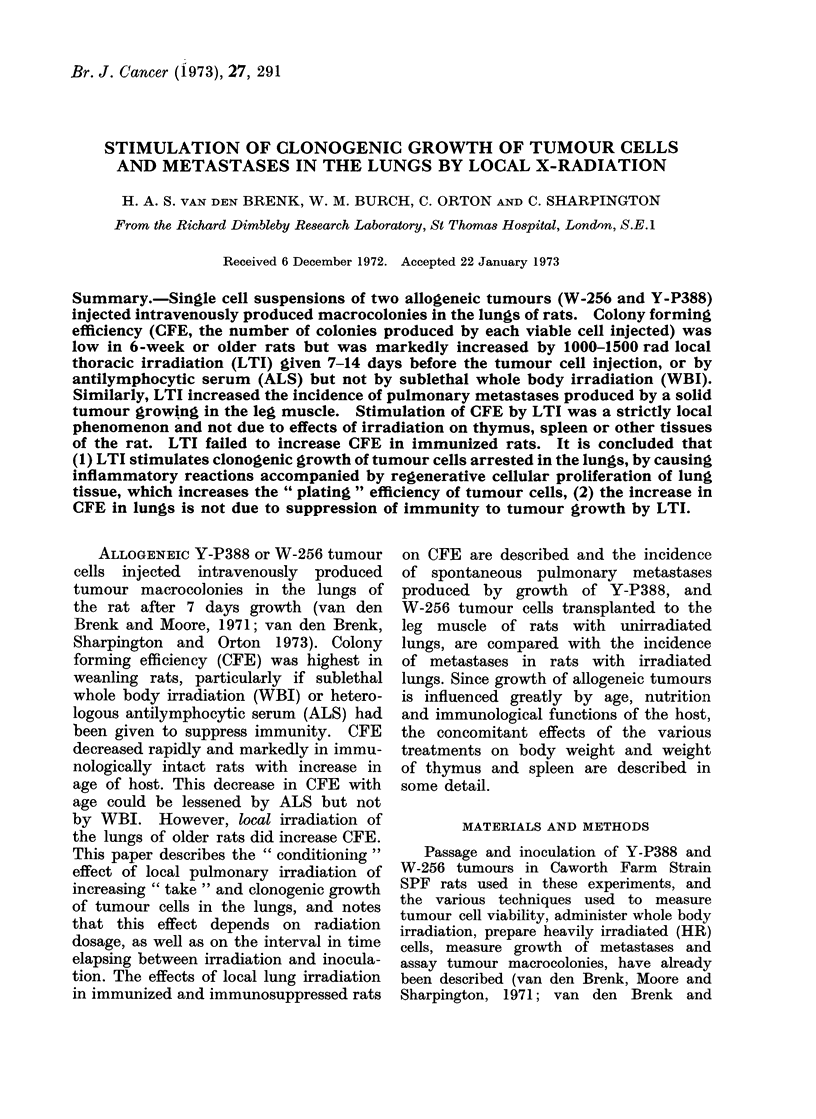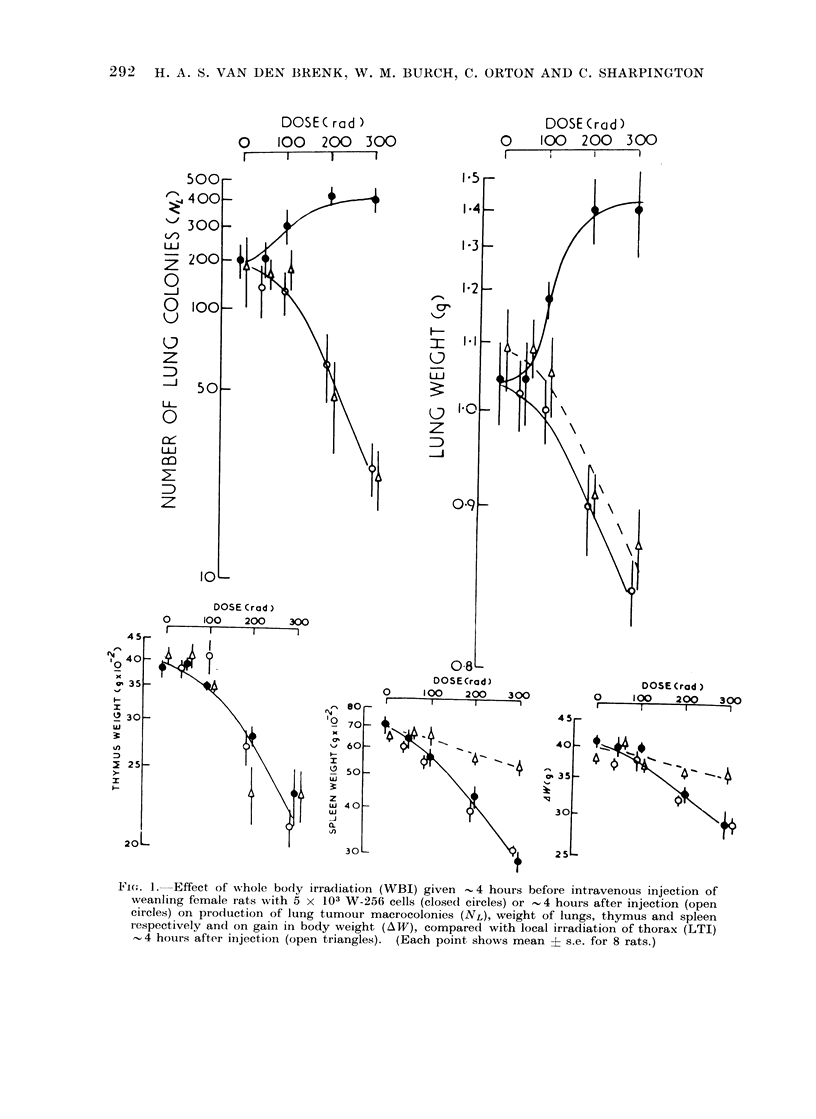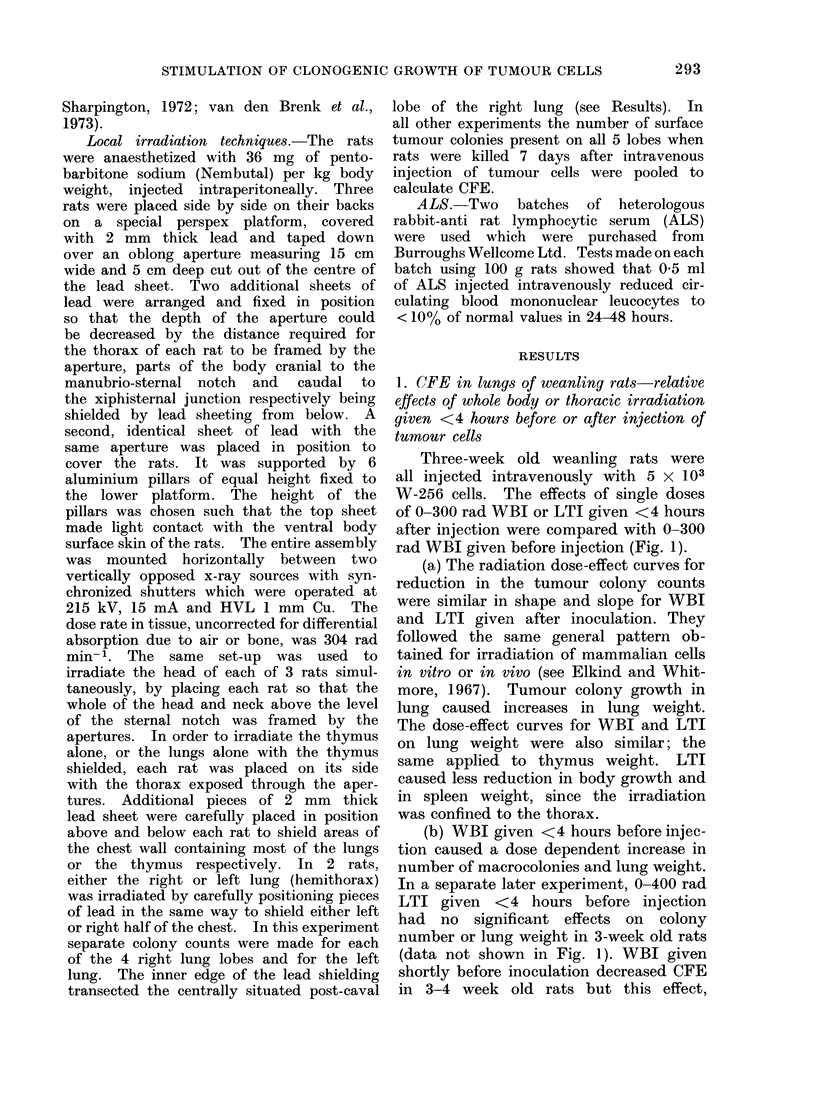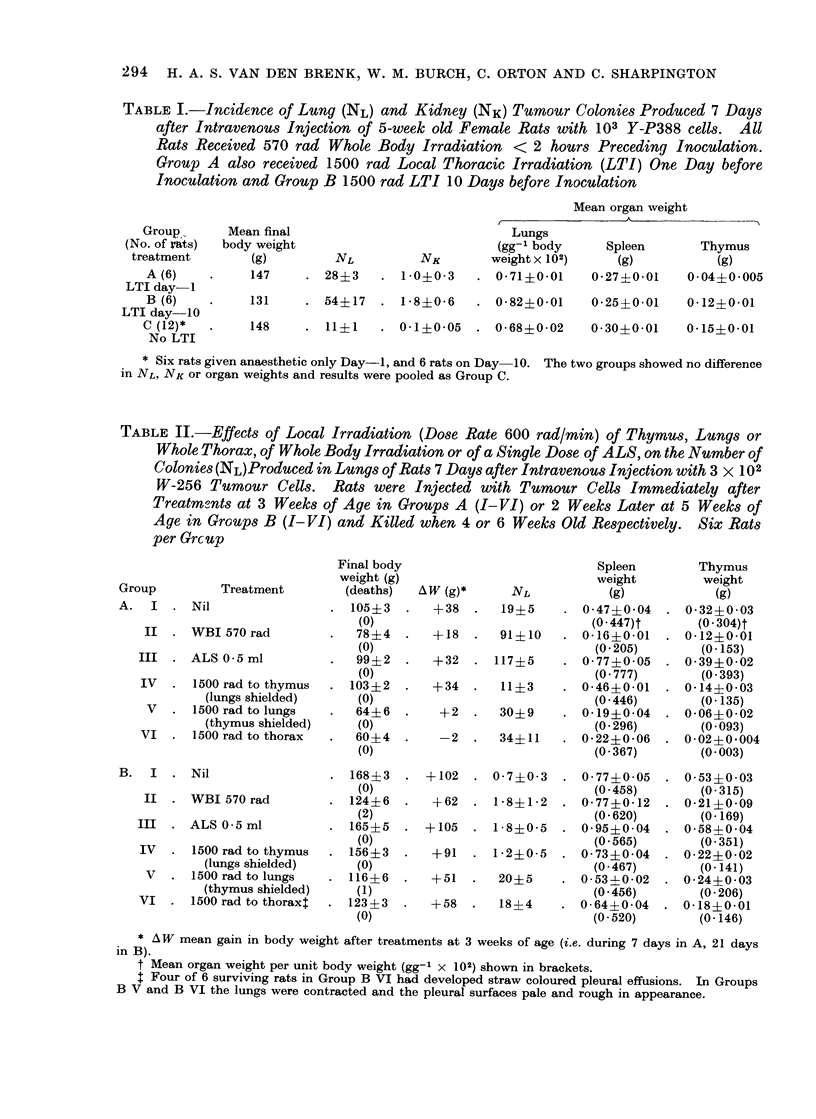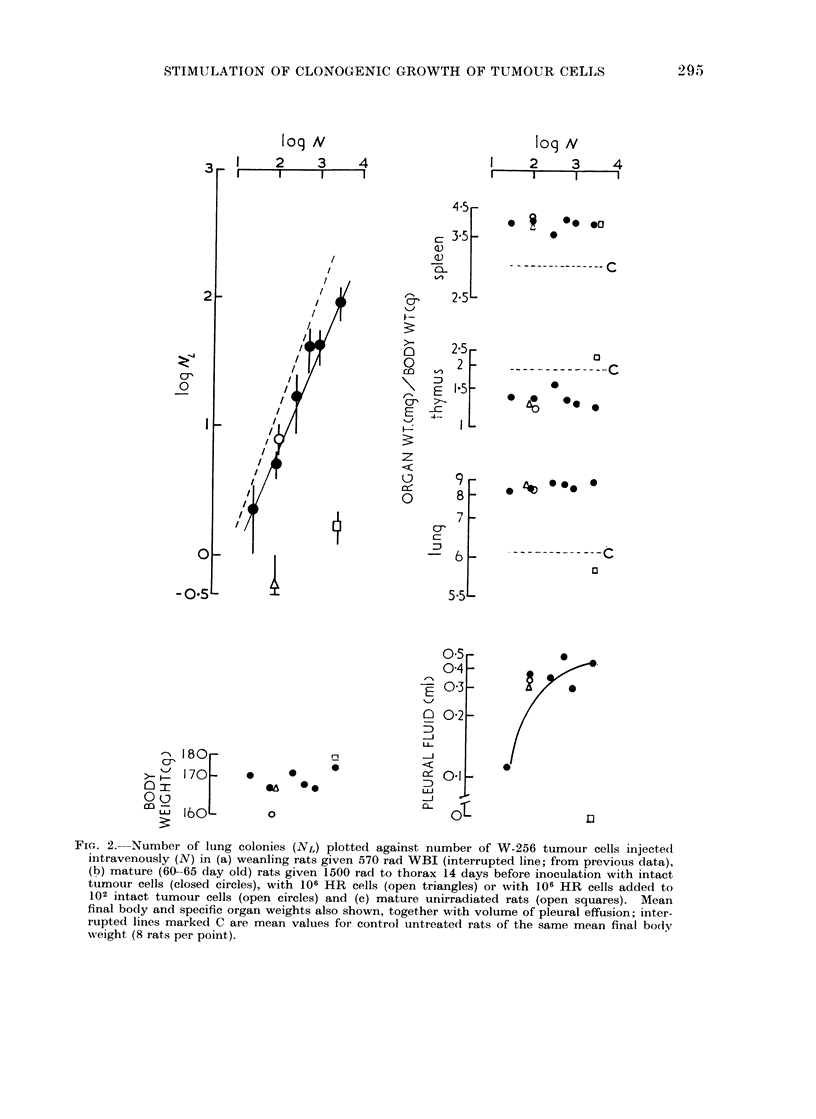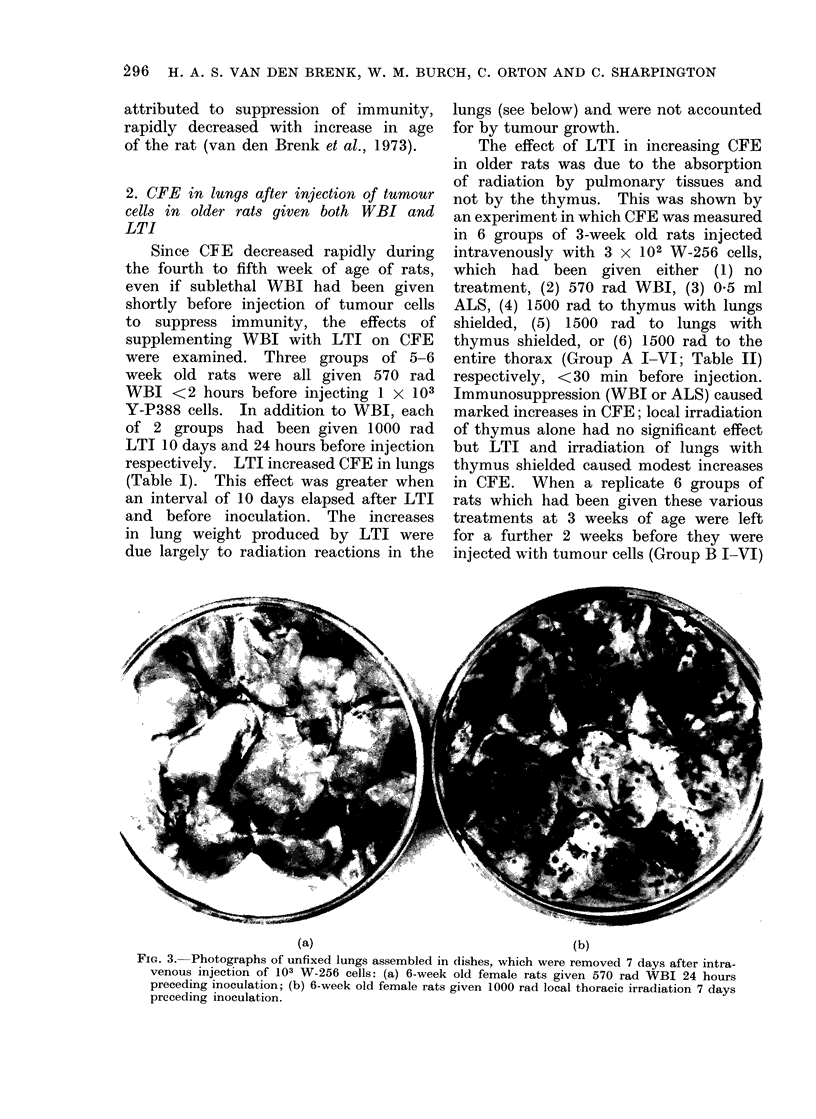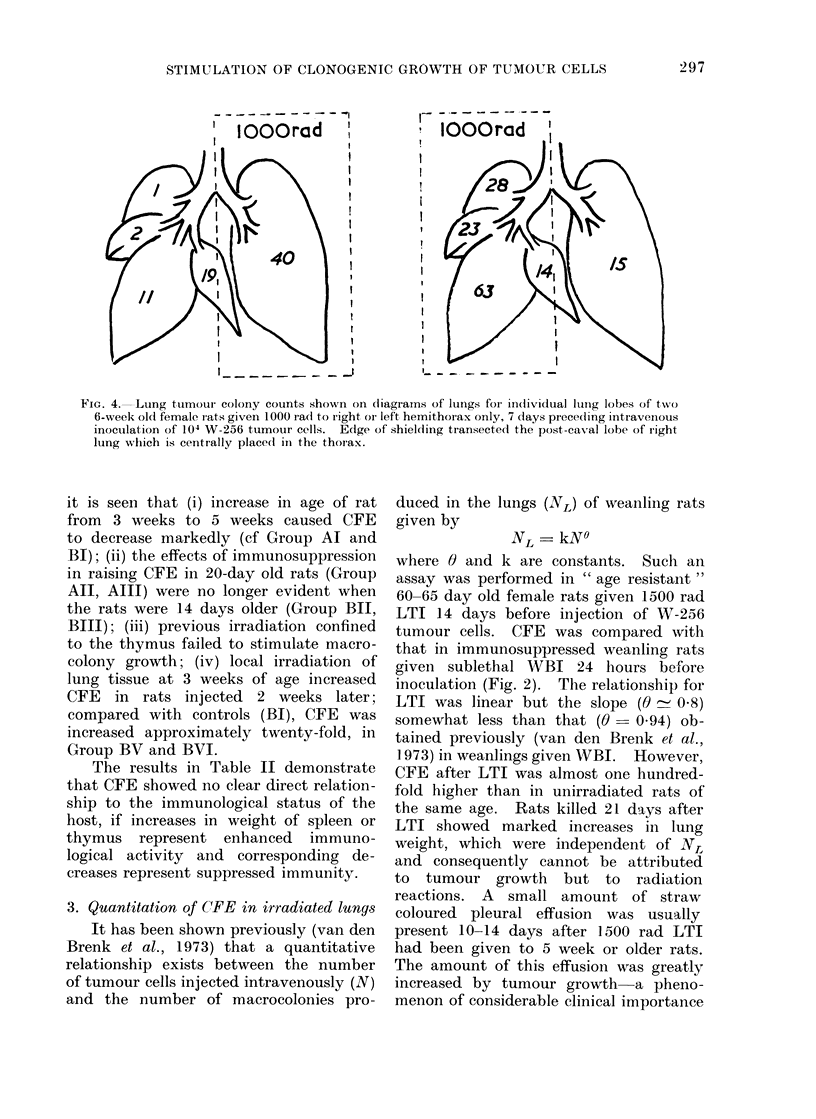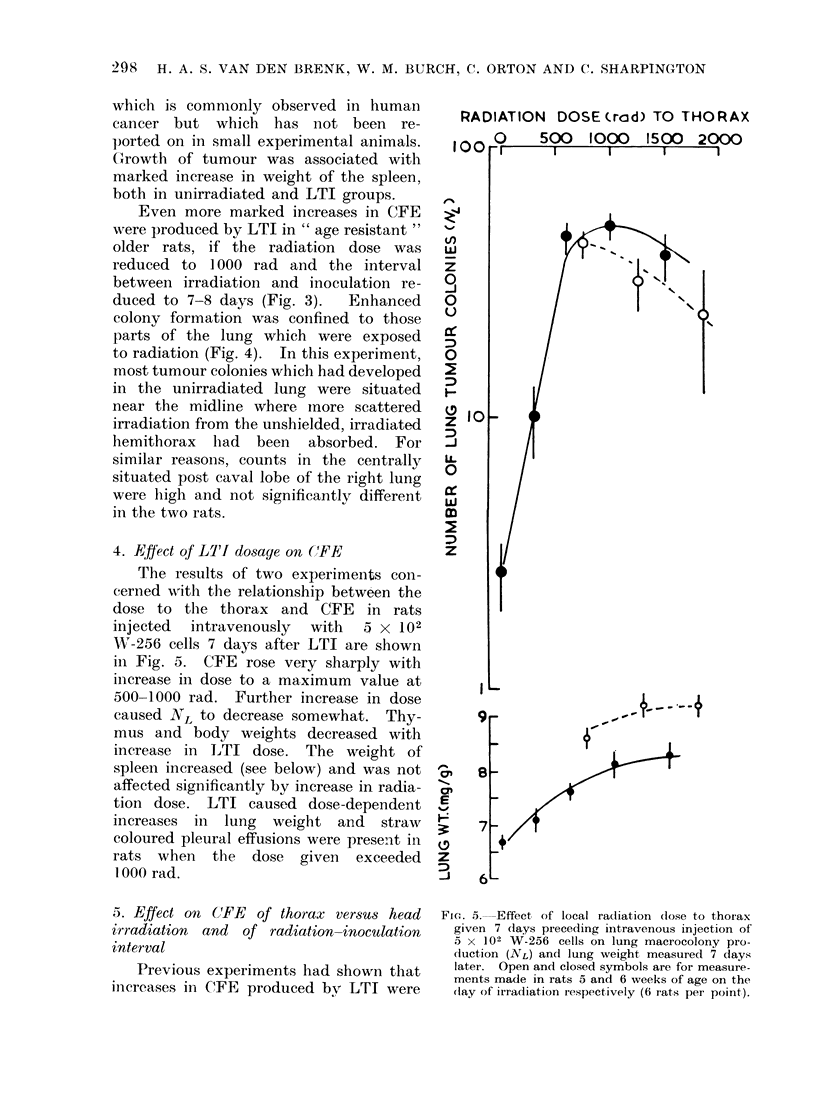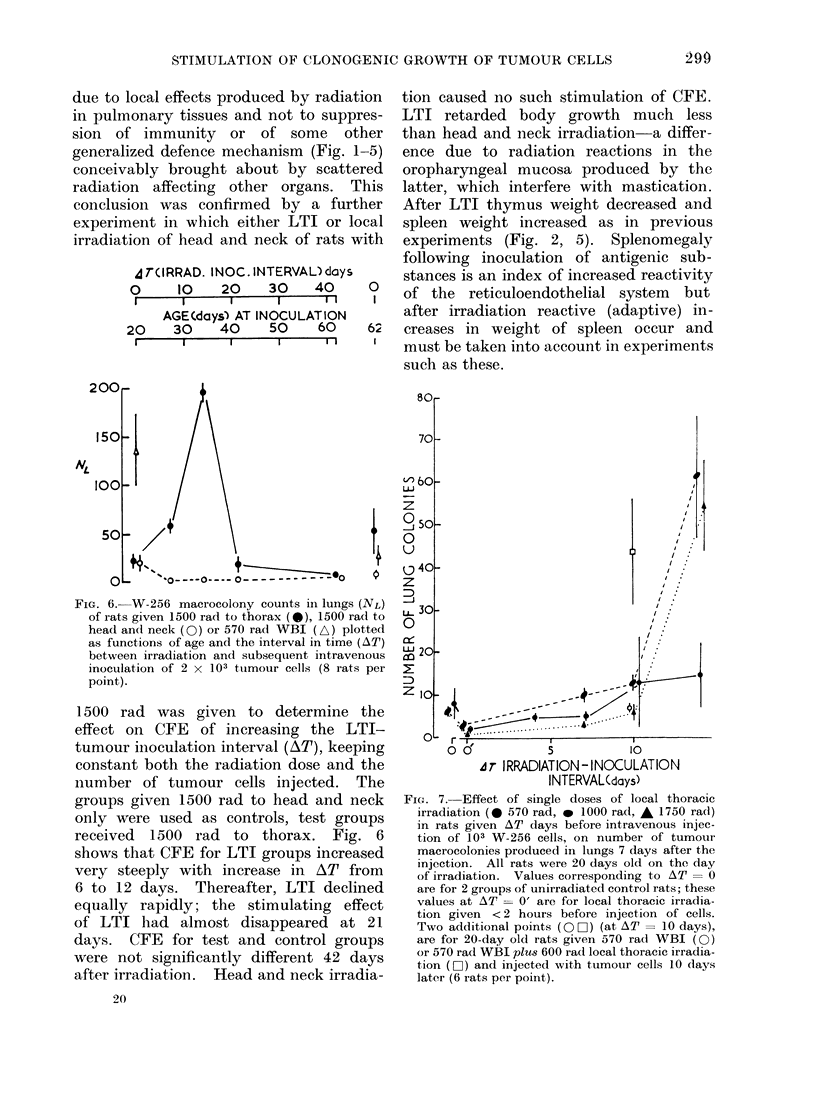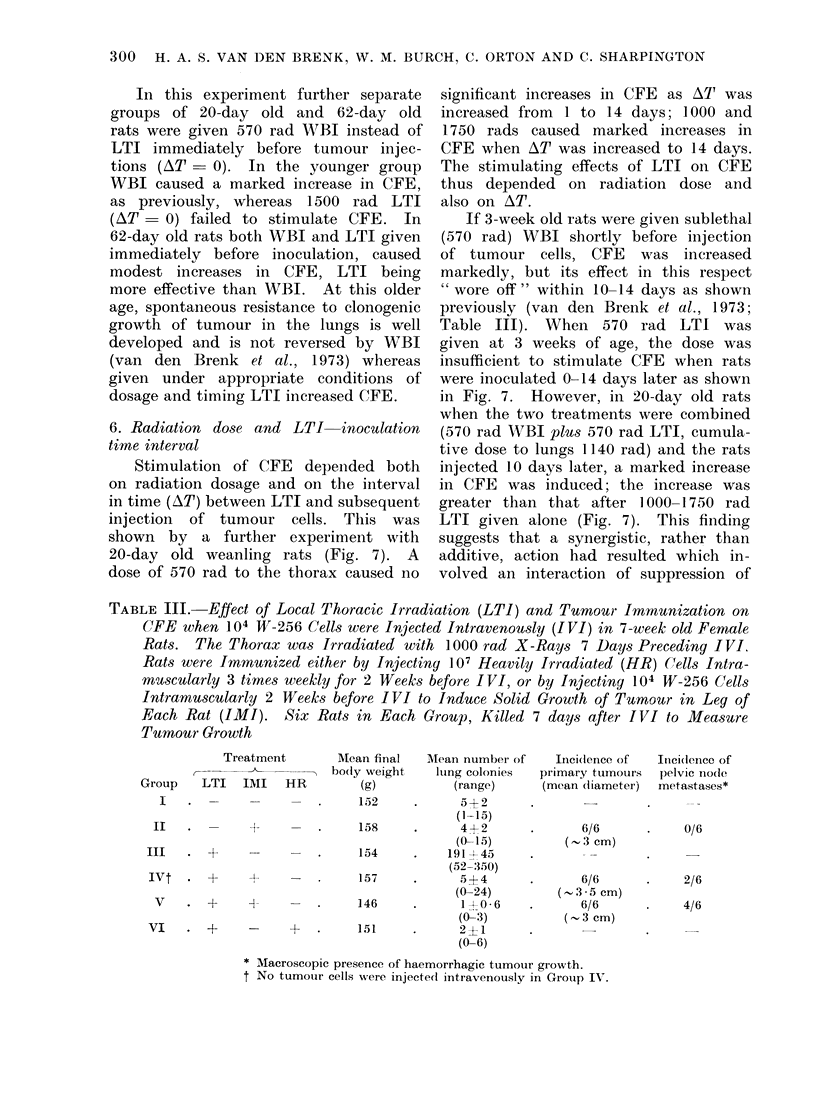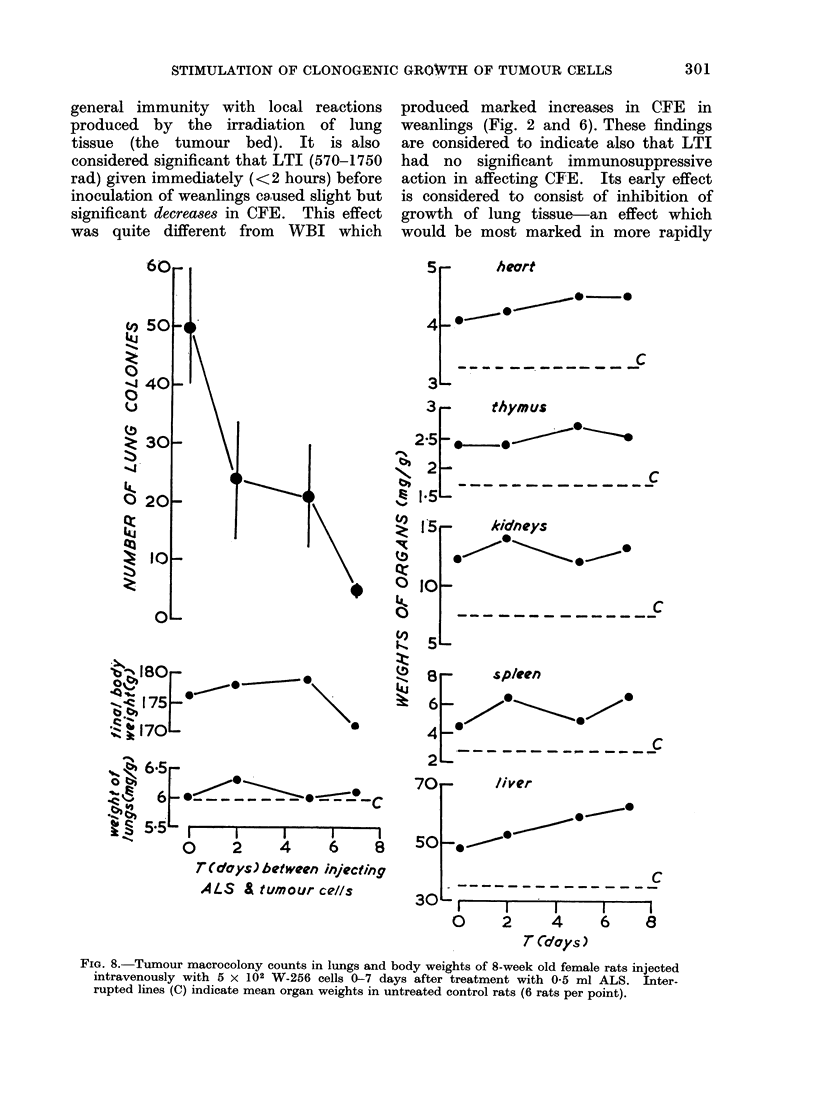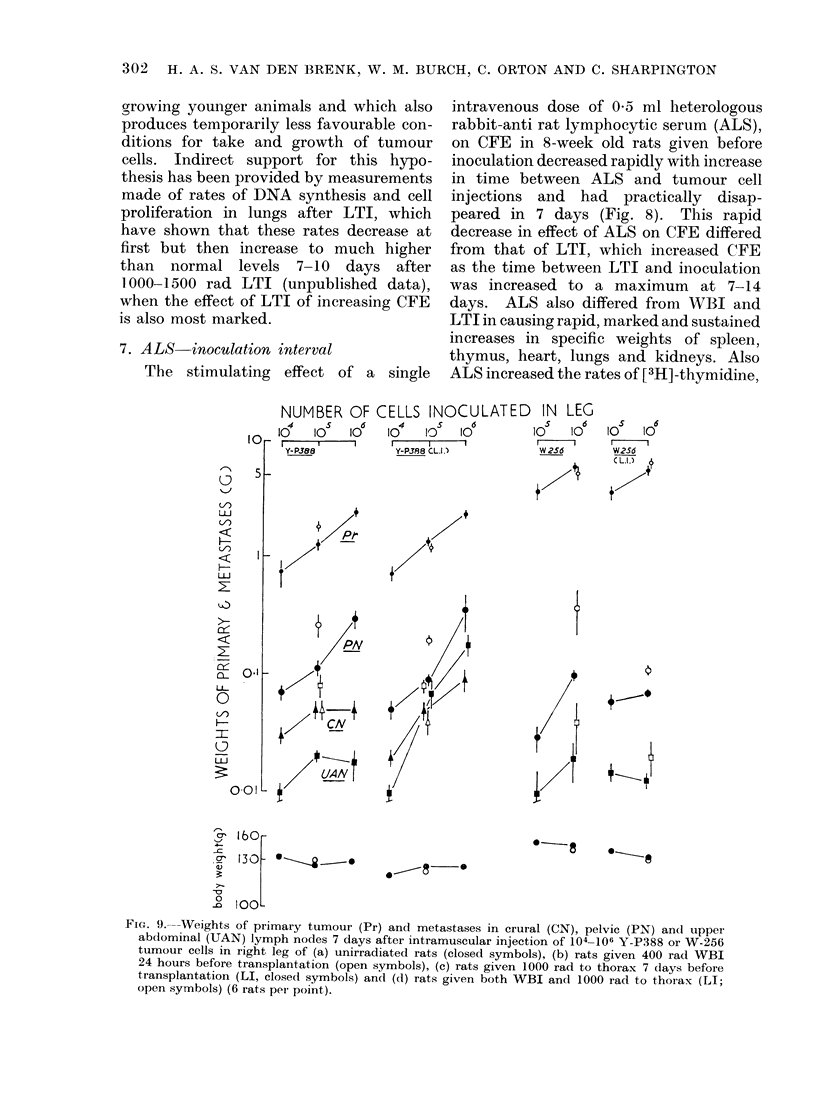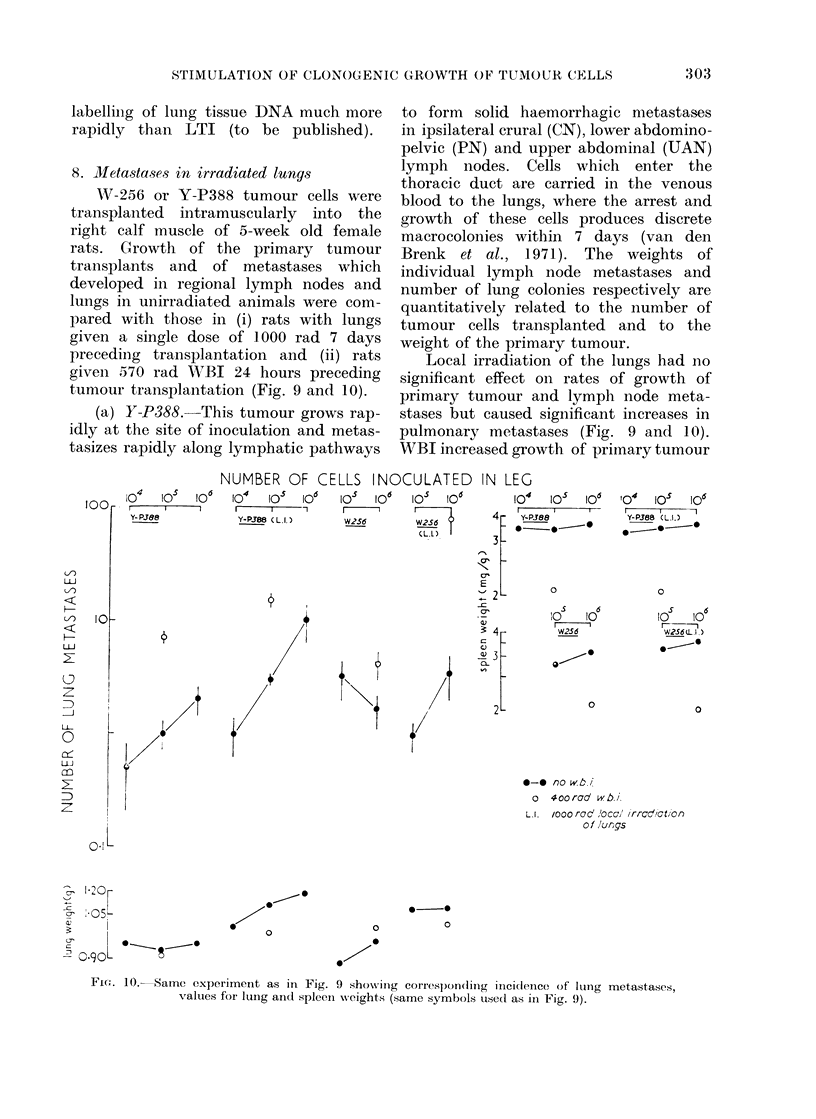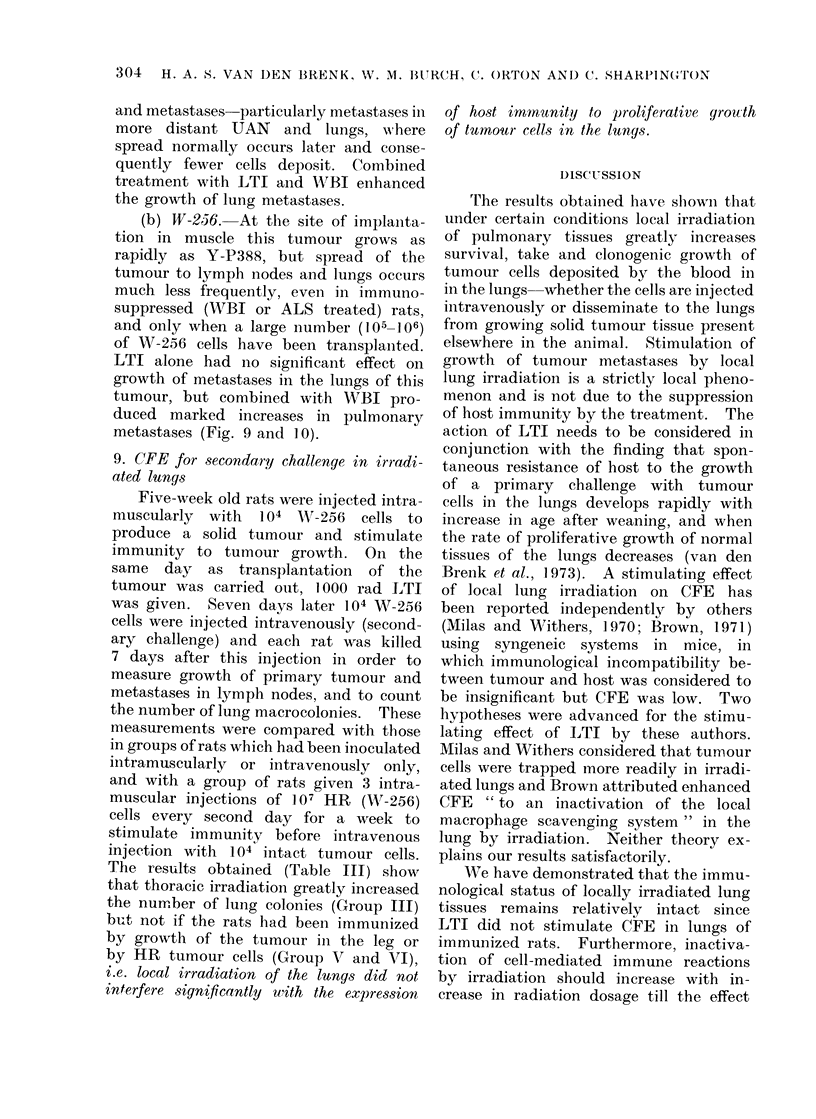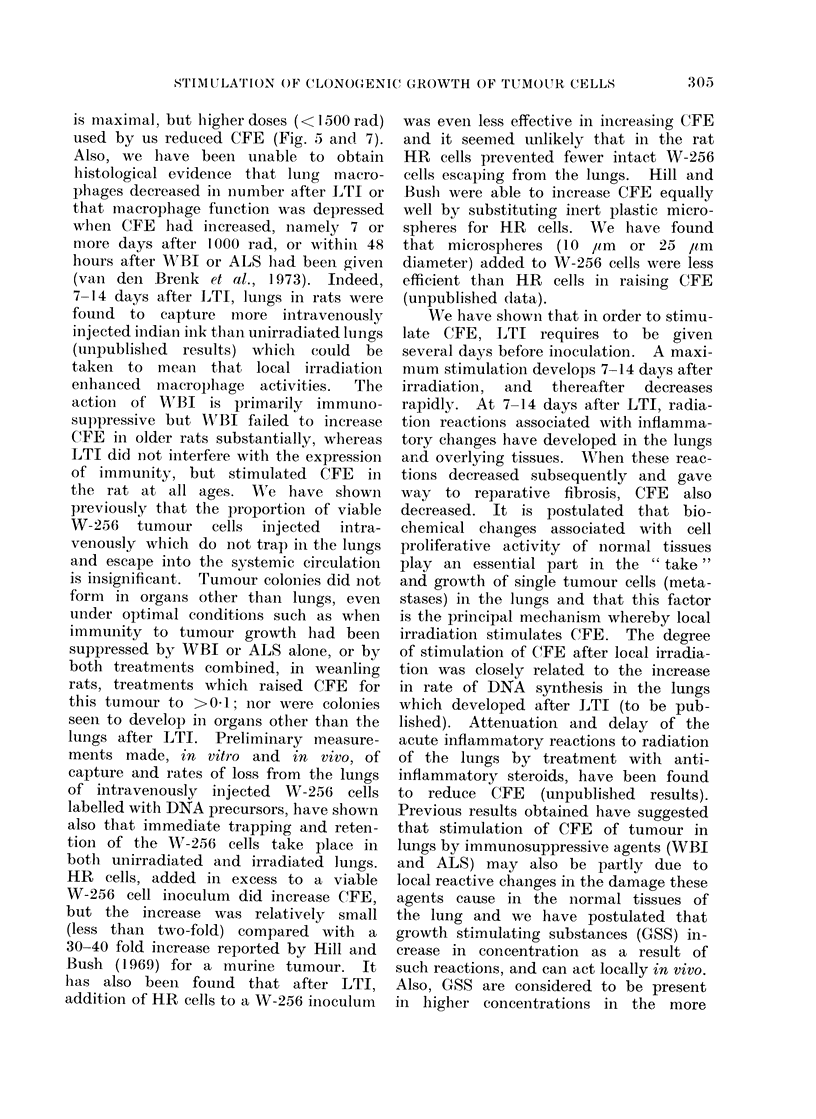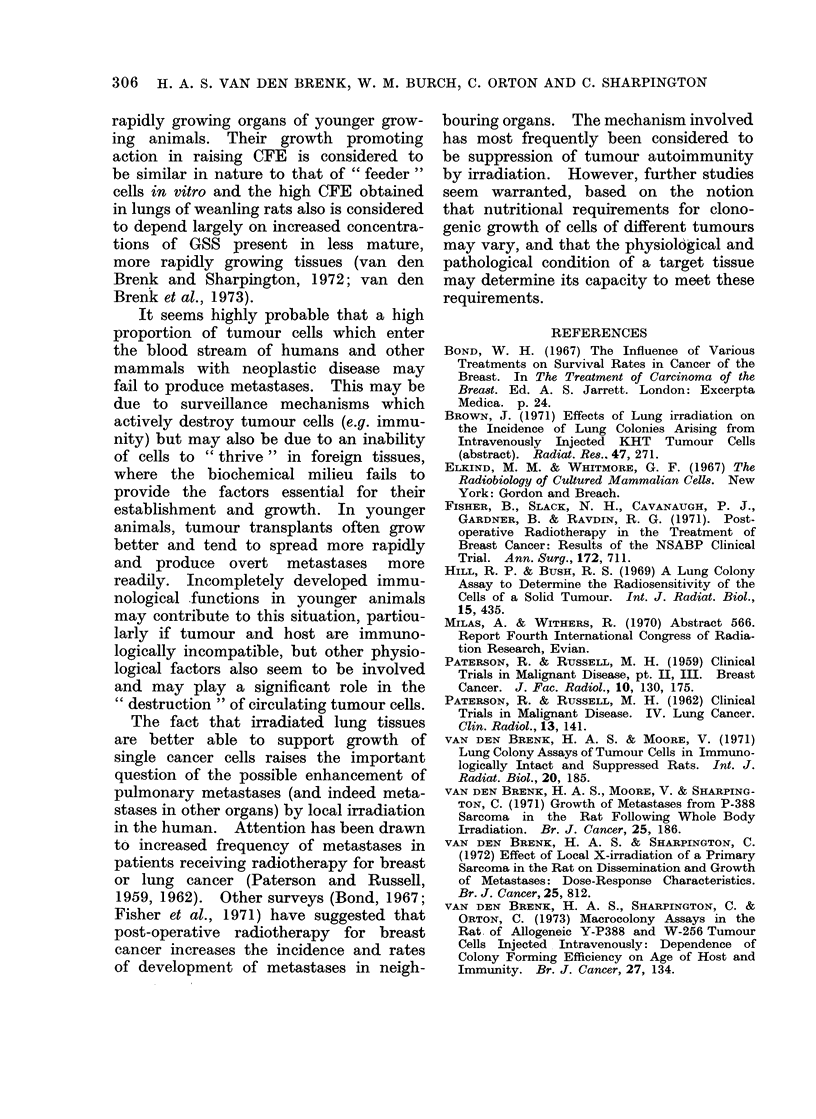# Stimulation of Clonogenic Growth of Tumour Cells and Metastases in the Lungs by Local X-Radiation

**DOI:** 10.1038/bjc.1973.36

**Published:** 1973-04

**Authors:** H. A. S. van den Brenk, W. M. Burch, C. Orton, C. Sharpington

## Abstract

**Images:**


*Br. J. Cancer* (1973), **27**, 291

### STIMULATION OF CLONOGENIC GROWTH OF TUMOUR CELLS AND METASTASES IN THE LUNGS BY LOCAL X-RADIATION

H. A. S. VAN DEN BRENK, W. M. BURCH, C. ORTON AND C. SHARPINGTON

*From the Richard Dimbleby Research Laboratory, St Thomas Hospital, London, S.E.*1

Received 6 December 1972. Accepted 22 January 1973

**Summary.—Single cell suspensions of two allogeneic tumours (W-256 and Y-P388) injected intravenously produced macrocolonies in the lungs of rats. Colony forming efficiency (CFE, the number of colonies produced by each viable cell injected) was low in 6-week or older rats but was markedly increased by 1000–1500 rad local thoracic irradiation (LTI) given 7–14 days before the tumour cell injection, or by antilymphocytic serum (ALS) but not by sublethal whole body irradiation (WBI). Similarly, LTI increased the incidence of pulmonary metastases produced by a solid tumour growing in the leg muscle. Stimulation of CFE by LTI was a strictly local phenomenon and not due to effects of irradiation on thymus, spleen or other tissues of the rat. LTI failed to increase CFE in immunized rats. It is concluded that (1) LTI stimulates clonogenic growth of tumour cells arrested in the lungs, by causing inflammatory reactions accompanied by regenerative cellular proliferation of lung tissue, which increases the " plating " efficiency of tumour cells, (2) the increase in CFE in lungs is not due to suppression of immunity to tumour growth by LTI.**

ALLOGENEIC Y-P388 or W-256 tumour cells injected intravenously produced tumour macrocolonies in the lungs of the rat after 7 days growth (van den Brenk and Moore, 1971; van den Brenk, Sharpington and Orton 1973). Colony forming efficiency (CFE) was highest in weanling rats, particularly if sublethal whole body irradiation (WBI) or heterologous antilymphocytic serum (ALS) had been given to suppress immunity. CFE decreased rapidly and markedly in immunologically intact rats with increase in age of host. This decrease in CFE with age could be lessened by ALS but not by WBI. However, *local* irradiation of the lungs of older rats did increase CFE. This paper describes the " conditioning " effect of local pulmonary irradiation of increasing " take " and clonogenic growth of tumour cells in the lungs, and notes that this effect depends on radiation dosage, as well as on the interval in time elapsing between irradiation and inoculation. The effects of local lung irradiation in immunized and immunosuppressed rats on CFE are described and the incidence of spontaneous pulmonary metastases produced by growth of Y-P388, and W-256 tumour cells transplanted to the leg muscle of rats with unirradiated lungs, are compared with the incidence of metastases in rats with irradiated lungs. Since growth of allogeneic tumours is influenced greatly by age, nutrition and immunological functions of the host, the concomitant effects of the various treatments on body weight and weight of thymus and spleen are described in some detail.

### MATERIALS AND METHODS

Passage and inoculation of Y-P388 and W-256 tumours in Caworth Farm Strain SPF rats used in these experiments, and the various techniques used to measure tumour cell viability, administer whole body irradiation, prepare heavily irradiated (HR) cells, measure growth of metastases and assay tumour macrocolonies, have already been described (van den Brenk, Moore and Sharpington, 1971; van den Brenk and

FIG. 1.—Effect of whole body irradiation (WBI) given ~4 hours before intravenous injection of weanling female rats with $5 \times 10^3$ W-256 cells (closed circles) or ~4 hours after injection (open circles) on production of lung tumour macrocolonies ($N_L$), weight of lungs, thymus and spleen respectively and on gain in body weight ($\Delta W$), compared with local irradiation of thorax (LTI) ~4 hours after injection (open triangles). (Each point shows mean $\pm$ s.e. for 8 rats.)

Sharpington, 1972; van den Brenk *et al.*, 1973).

*Local irradiation techniques.*—The rats were anaesthetized with 36 mg of pentobarbitone sodium (Nembutal) per kg body weight, injected intraperitoneally. Three rats were placed side by side on their backs on a special perspex platform, covered with 2 mm thick lead and taped down over an oblong aperture measuring 15 cm wide and 5 cm deep cut out of the centre of the lead sheet. Two additional sheets of lead were arranged and fixed in position so that the depth of the aperture could be decreased by the distance required for the thorax of each rat to be framed by the aperture, parts of the body cranial to the manubrio-sternal notch and caudal to the xiphisternal junction respectively being shielded by lead sheeting from below. A second, identical sheet of lead with the same aperture was placed in position to cover the rats. It was supported by 6 aluminium pillars of equal height fixed to the lower platform. The height of the pillars was chosen such that the top sheet made light contact with the ventral body surface skin of the rats. The entire assembly was mounted horizontally between two vertically opposed x-ray sources with synchronized shutters which were operated at 215 kV, 15 mA and HVL 1 mm Cu. The dose rate in tissue, uncorrected for differential absorption due to air or bone, was 304 rad $min^{-1}$. The same set-up was used to irradiate the head of each of 3 rats simultaneously, by placing each rat so that the whole of the head and neck above the level of the sternal notch was framed by the apertures. In order to irradiate the thymus alone, or the lungs alone with the thymus shielded, each rat was placed on its side with the thorax exposed through the apertures. Additional pieces of 2 mm thick lead sheet were carefully placed in position above and below each rat to shield areas of the chest wall containing most of the lungs or the thymus respectively. In 2 rats, either the right or left lung (hemithorax) was irradiated by carefully positioning pieces of lead in the same way to shield either left or right half of the chest. In this experiment separate colony counts were made for each of the 4 right lung lobes and for the left lung. The inner edge of the lead shielding transected the centrally situated post-caval lobe of the right lung (see Results). In all other experiments the number of surface tumour colonies present on all 5 lobes when rats were killed 7 days after intravenous injection of tumour cells were pooled to calculate CFE.

*ALS.*—Two batches of heterologous rabbit-anti rat lymphocytic serum (ALS) were used which were purchased from Burroughs Wellcome Ltd. Tests made on each batch using 100 g rats showed that 0·5 ml of ALS injected intravenously reduced circulating blood mononuclear leucocytes to $<10\%$ of normal values in 24–48 hours.

### RESULTS

### 1. *CFE in lungs of weanling rats—relative effects of whole body or thoracic irradiation given <4 hours before or after injection of tumour cells*

Three-week old weanling rats were all injected intravenously with $5 \times 10^3$ W-256 cells. The effects of single doses of 0–300 rad WBI or LTI given <4 hours after injection were compared with 0–300 rad WBI given before injection (Fig. 1).

(a) The radiation dose-effect curves for reduction in the tumour colony counts were similar in shape and slope for WBI and LTI given after inoculation. They followed the same general pattern obtained for irradiation of mammalian cells *in vitro* or *in vivo* (see Elkind and Whitmore, 1967). Tumour colony growth in lung caused increases in lung weight. The dose-effect curves for WBI and LTI on lung weight were also similar; the same applied to thymus weight. LTI caused less reduction in body growth and in spleen weight, since the irradiation was confined to the thorax.

(b) WBI given <4 hours before injection caused a dose dependent increase in number of macrocolonies and lung weight. In a separate later experiment, 0–400 rad LTI given <4 hours before injection had no significant effects on colony number or lung weight in 3-week old rats (data not shown in Fig. 1). WBI given shortly before inoculation decreased CFE in 3–4 week old rats but this effect,

TABLE I.—*Incidence of Lung* ($N_L$) *and Kidney* ($N_K$) *Tumour Colonies Produced* 7 *Days after Intravenous Injection of* 5-*week old Female Rats with* $10^3$ *Y-P*388 *cells. All Rats Received* 570 *rad Whole Body Irradiation* < 2 *hours Preceding Inoculation. Group A also received* 1500 *rad Local Thoracic Irradiation* (*LTI*) *One Day before Inoculation and Group B* 1500 *rad LTI* 10 *Days before Inoculation*

| Group (No. of rats) treatment | Mean final body weight (g) | $N_L$ | $N_K$ | Mean organ weight: Lungs (gg$^{-1}$ body weight × $10^2$) | Spleen (g) | Thymus (g) |
|---|---|---|---|---|---|---|
| A (6) LTI day—1 | 147 | 28±3 | 1·0±0·3 | 0·71±0·01 | 0·27±0·01 | 0·04±0·005 |
| B (6) LTI day—10 | 131 | 54±17 | 1·8±0·6 | 0·82±0·01 | 0·25±0·01 | 0·12±0·01 |
| C (12)* No LTI | 148 | 11±1 | 0·1±0·05 | 0·68±0·02 | 0·30±0·01 | 0·15±0·01 |

* Six rats given anaesthetic only Day—1, and 6 rats on Day—10. The two groups showed no difference in $N_L$, $N_K$ or organ weights and results were pooled as Group C.

TABLE II.—*Effects of Local Irradiation* (*Dose Rate* 600 *rad/min*) *of Thymus, Lungs or Whole Thorax, of Whole Body Irradiation or of a Single Dose of ALS, on the Number of Colonies* ($N_L$) *Produced in Lungs of Rats* 7 *Days after Intravenous Injection with* 3 × $10^2$ *W*-256 *Tumour Cells. Rats were Injected with Tumour Cells Immediately after Treatments at* 3 *Weeks of Age in Groups A* (*I–VI*) *or* 2 *Weeks Later at* 5 *Weeks of Age in Groups B* (*I–VI*) *and Killed when* 4 *or* 6 *Weeks Old Respectively. Six Rats per Group*

| Group | Treatment | Final body weight (g) (deaths) | $\Delta W$ (g)* | $N_L$ | Spleen weight (g) | Thymus weight (g) |
|---|---|---|---|---|---|---|
| A. I | Nil | 105±3 (0) | +38 | 19±5 | 0·47±0·04 (0·447)† | 0·32±0·03 (0·304)† |
| II | WBI 570 rad | 78±4 (0) | +18 | 91±10 | 0·16±0·01 (0·205) | 0·12±0·01 (0·153) |
| III | ALS 0·5 ml | 99±2 (0) | +32 | 117±5 | 0·77±0·05 (0·777) | 0·39±0·02 (0·393) |
| IV | 1500 rad to thymus (lungs shielded) | 103±2 (0) | +34 | 11±3 | 0·46±0·01 (0·446) | 0·14±0·03 (0·135) |
| V | 1500 rad to lungs (thymus shielded) | 64±6 (0) | +2 | 30±9 | 0·19±0·04 (0·296) | 0·06±0·02 (0·093) |
| VI | 1500 rad to thorax | 60±4 (0) | −2 | 34±11 | 0·22±0·06 (0·367) | 0·02±0·004 (0·003) |
| B. I | Nil | 168±3 (0) | +102 | 0·7±0·3 | 0·77±0·05 (0·458) | 0·53±0·03 (0·315) |
| II | WBI 570 rad | 124±6 (2) | +62 | 1·8±1·2 | 0·77±0·12 (0·620) | 0·21±0·09 (0·169) |
| III | ALS 0·5 ml | 165±5 (0) | +105 | 1·8±0·5 | 0·95±0·04 (0·565) | 0·58±0·04 (0·351) |
| IV | 1500 rad to thymus (lungs shielded) | 156±3 (0) | +91 | 1·2±0·5 | 0·73±0·04 (0·467) | 0·22±0·02 (0·141) |
| V | 1500 rad to lungs (thymus shielded) | 116±6 (1) | +51 | 20±5 | 0·53±0·02 (0·456) | 0·24±0·03 (0·206) |
| VI | 1500 rad to thorax‡ | 123±3 (0) | +58 | 18±4 | 0·64±0·04 (0·520) | 0·18±0·01 (0·146) |

* $\Delta W$ mean gain in body weight after treatments at 3 weeks of age (*i.e.* during 7 days in A, 21 days in B).

† Mean organ weight per unit body weight (gg$^{-1}$ × $10^2$) shown in brackets.

‡ Four of 6 surviving rats in Group B VI had developed straw coloured pleural effusions. In Groups B V and B VI the lungs were contracted and the pleural surfaces pale and rough in appearance.

FIG. 2.—Number of lung colonies ($N_L$) plotted against number of W-256 tumour cells injected intravenously ($N$) in (a) weanling rats given 570 rad WBI (interrupted line; from previous data), (b) mature (60–65 day old) rats given 1500 rad to thorax 14 days before inoculation with intact tumour cells (closed circles), with $10^6$ HR cells (open triangles) or with $10^6$ HR cells added to $10^2$ intact tumour cells (open circles) and (c) mature unirradiated rats (open squares). Mean final body and specific organ weights also shown, together with volume of pleural effusion; interrupted lines marked C are mean values for control untreated rats of the same mean final body weight (8 rats per point).

attributed to suppression of immunity, rapidly decreased with increase in age of the rat (van den Brenk *et al.*, 1973).

### 2. *CFE in lungs after injection of tumour cells in older rats given both WBI and LTI*

Since CFE decreased rapidly during the fourth to fifth week of age of rats, even if sublethal WBI had been given shortly before injection of tumour cells to suppress immunity, the effects of supplementing WBI with LTI on CFE were examined. Three groups of 5–6 week old rats were all given 570 rad WBI <2 hours before injecting $1 \times 10^3$ Y-P388 cells. In addition to WBI, each of 2 groups had been given 1000 rad LTI 10 days and 24 hours before injection respectively. LTI increased CFE in lungs (Table I). This effect was greater when an interval of 10 days elapsed after LTI and before inoculation. The increases in lung weight produced by LTI were due largely to radiation reactions in the lungs (see below) and were not accounted for by tumour growth.

The effect of LTI in increasing CFE in older rats was due to the absorption of radiation by pulmonary tissues and not by the thymus. This was shown by an experiment in which CFE was measured in 6 groups of 3-week old rats injected intravenously with $3 \times 10^2$ W-256 cells, which had been given either (1) no treatment, (2) 570 rad WBI, (3) 0·5 ml ALS, (4) 1500 rad to thymus with lungs shielded, (5) 1500 rad to lungs with thymus shielded, or (6) 1500 rad to the entire thorax (Group A I–VI; Table II) respectively, <30 min before injection. Immunosuppression (WBI or ALS) caused marked increases in CFE; local irradiation of thymus alone had no significant effect but LTI and irradiation of lungs with thymus shielded caused modest increases in CFE. When a replicate 6 groups of rats which had been given these various treatments at 3 weeks of age were left for a further 2 weeks before they were injected with tumour cells (Group B I–VI)

FIG. 3.—Photographs of unfixed lungs assembled in dishes, which were removed 7 days after intravenous injection of $10^3$ W-256 cells: (a) 6-week old female rats given 570 rad WBI 24 hours preceding inoculation; (b) 6-week old female rats given 1000 rad local thoracic irradiation 7 days preceding inoculation.

Fig. 4.—Lung tumour colony counts shown on diagrams of lungs for individual lung lobes of two 6-week old female rats given 1000 rad to right or left hemithorax only, 7 days preceding intravenous inoculation of $10^4$ W-256 tumour cells. Edge of shielding transected the post-caval lobe of right lung which is centrally placed in the thorax.

it is seen that (i) increase in age of rat from 3 weeks to 5 weeks caused CFE to decrease markedly (cf Group AI and BI); (ii) the effects of immunosuppression in raising CFE in 20-day old rats (Group AII, AIII) were no longer evident when the rats were 14 days older (Group BII, BIII); (iii) previous irradiation confined to the thymus failed to stimulate macrocolony growth; (iv) local irradiation of lung tissue at 3 weeks of age increased CFE in rats injected 2 weeks later; compared with controls (BI), CFE was increased approximately twenty-fold, in Group BV and BVI.

The results in Table II demonstrate that CFE showed no clear direct relationship to the immunological status of the host, if increases in weight of spleen or thymus represent enhanced immunological activity and corresponding decreases represent suppressed immunity.

3. *Quantitation of CFE in irradiated lungs*

It has been shown previously (van den Brenk *et al.*, 1973) that a quantitative relationship exists between the number of tumour cells injected intravenously ($N$) and the number of macrocolonies produced in the lungs ($N_L$) of weanling rats given by

$$N_L = \mathrm{k}N^{\theta}$$

where $\theta$ and k are constants. Such an assay was performed in "age resistant" 60–65 day old female rats given 1500 rad LTI 14 days before injection of W-256 tumour cells. CFE was compared with that in immunosuppressed weanling rats given sublethal WBI 24 hours before inoculation (Fig. 2). The relationship for LTI was linear but the slope ($\theta \simeq 0{\cdot}8$) somewhat less than that ($\theta = 0{\cdot}94$) obtained previously (van den Brenk *et al.*, 1973) in weanlings given WBI. However, CFE after LTI was almost one hundred-fold higher than in unirradiated rats of the same age. Rats killed 21 days after LTI showed marked increases in lung weight, which were independent of $N_L$ and consequently cannot be attributed to tumour growth but to radiation reactions. A small amount of straw coloured pleural effusion was usually present 10–14 days after 1500 rad LTI had been given to 5 week or older rats. The amount of this effusion was greatly increased by tumour growth—a phenomenon of considerable clinical importance which is commonly observed in human cancer but which has not been reported on in small experimental animals. Growth of tumour was associated with marked increase in weight of the spleen, both in unirradiated and LTI groups.

Even more marked increases in CFE were produced by LTI in " age resistant " older rats, if the radiation dose was reduced to 1000 rad and the interval between irradiation and inoculation reduced to 7–8 days (Fig. 3). Enhanced colony formation was confined to those parts of the lung which were exposed to radiation (Fig. 4). In this experiment, most tumour colonies which had developed in the unirradiated lung were situated near the midline where more scattered irradiation from the unshielded, irradiated hemithorax had been absorbed. For similar reasons, counts in the centrally situated post caval lobe of the right lung were high and not significantly different in the two rats.

4. *Effect of LTI dosage on CFE*

The results of two experiments concerned with the relationship between the dose to the thorax and CFE in rats injected intravenously with $5 \times 10^2$ W-256 cells 7 days after LTI are shown in Fig. 5. CFE rose very sharply with increase in dose to a maximum value at 500–1000 rad. Further increase in dose caused $N_L$ to decrease somewhat. Thymus and body weights decreased with increase in LTI dose. The weight of spleen increased (see below) and was not affected significantly by increase in radiation dose. LTI caused dose-dependent increases in lung weight and straw coloured pleural effusions were present in rats when the dose given exceeded 1000 rad.

Fig. 5.—Effect of local radiation dose to thorax given 7 days preceding intravenous injection of $5 \times 10^2$ W-256 cells on lung macrocolony production ($N_L$) and lung weight measured 7 days later. Open and closed symbols are for measurements made in rats 5 and 6 weeks of age on the day of irradiation respectively (6 rats per point).

5. *Effect on CFE of thorax versus head irradiation and of radiation–inoculation interval*

Previous experiments had shown that increases in CFE produced by LTI were due to local effects produced by radiation in pulmonary tissues and not to suppression of immunity or of some other generalized defence mechanism (Fig. 1–5) conceivably brought about by scattered radiation affecting other organs. This conclusion was confirmed by a further experiment in which either LTI or local irradiation of head and neck of rats with

Fig. 6.—W-256 macrocolony counts in lungs ($N_L$) of rats given 1500 rad to thorax (●), 1500 rad to head and neck (○) or 570 rad WBI (△) plotted as functions of age and the interval in time ($\Delta T$) between irradiation and subsequent intravenous inoculation of $2 \times 10^3$ tumour cells (8 rats per point).

1500 rad was given to determine the effect on CFE of increasing the LTI–tumour inoculation interval ($\Delta T$), keeping constant both the radiation dose and the number of tumour cells injected. The groups given 1500 rad to head and neck only were used as controls, test groups received 1500 rad to thorax. Fig. 6 shows that CFE for LTI groups increased very steeply with increase in $\Delta T$ from 6 to 12 days. Thereafter, LTI declined equally rapidly; the stimulating effect of LTI had almost disappeared at 21 days. CFE for test and control groups were not significantly different 42 days after irradiation. Head and neck irradiation caused no such stimulation of CFE. LTI retarded body growth much less than head and neck irradiation—a difference due to radiation reactions in the oropharyngeal mucosa produced by the latter, which interfere with mastication. After LTI thymus weight decreased and spleen weight increased as in previous experiments (Fig. 2, 5). Splenomegaly following inoculation of antigenic substances is an index of increased reactivity of the reticuloendothelial system but after irradiation reactive (adaptive) increases in weight of spleen occur and must be taken into account in experiments such as these.

Fig. 7.—Effect of single doses of local thoracic irradiation (● 570 rad, ● 1000 rad, ▲ 1750 rad) in rats given $\Delta T$ days before intravenous injection of $10^3$ W-256 cells, on number of tumour macrocolonies produced in lungs 7 days after the injection. All rats were 20 days old on the day of irradiation. Values corresponding to $\Delta T = 0$ are for 2 groups of unirradiated control rats; these values at $\Delta T = 0'$ are for local thoracic irradiation given $<2$ hours before injection of cells. Two additional points (○□) (at $\Delta T = 10$ days), are for 20-day old rats given 570 rad WBI (○) or 570 rad WBI *plus* 600 rad local thoracic irradiation (□) and injected with tumour cells 10 days later (6 rats per point).

In this experiment further separate groups of 20-day old and 62-day old rats were given 570 rad WBI instead of LTI immediately before tumour injections ($\Delta T = 0$). In the younger group WBI caused a marked increase in CFE, as previously, whereas 1500 rad LTI ($\Delta T = 0$) failed to stimulate CFE. In 62-day old rats both WBI and LTI given immediately before inoculation, caused modest increases in CFE, LTI being more effective than WBI. At this older age, spontaneous resistance to clonogenic growth of tumour in the lungs is well developed and is not reversed by WBI (van den Brenk *et al.*, 1973) whereas given under appropriate conditions of dosage and timing LTI increased CFE.

6. *Radiation dose and LTI—inoculation time interval*

Stimulation of CFE depended both on radiation dosage and on the interval in time ($\Delta T$) between LTI and subsequent injection of tumour cells. This was shown by a further experiment with 20-day old weanling rats (Fig. 7). A dose of 570 rad to the thorax caused no significant increases in CFE as $\Delta T$ was increased from 1 to 14 days; 1000 and 1750 rads caused marked increases in CFE when $\Delta T$ was increased to 14 days. The stimulating effects of LTI on CFE thus depended on radiation dose and also on $\Delta T$.

If 3-week old rats were given sublethal (570 rad) WBI shortly before injection of tumour cells, CFE was increased markedly, but its effect in this respect " wore off " within 10–14 days as shown previously (van den Brenk *et al.*, 1973; Table III). When 570 rad LTI was given at 3 weeks of age, the dose was insufficient to stimulate CFE when rats were inoculated 0–14 days later as shown in Fig. 7. However, in 20-day old rats when the two treatments were combined (570 rad WBI *plus* 570 rad LTI, cumulative dose to lungs 1140 rad) and the rats injected 10 days later, a marked increase in CFE was induced; the increase was greater than that after 1000–1750 rad LTI given alone (Fig. 7). This finding suggests that a synergistic, rather than additive, action had resulted which involved an interaction of suppression of

TABLE III.—*Effect of Local Thoracic Irradiation (LTI) and Tumour Immunization on CFE when* $10^4$ *W-256 Cells were Injected Intravenously (IVI) in 7-week old Female Rats. The Thorax was Irradiated with* 1000 *rad X-Rays* 7 *Days Preceding IVI. Rats were Immunized either by Injecting* $10^7$ *Heavily Irradiated (HR) Cells Intramuscularly* 3 *times weekly for* 2 *Weeks before IVI, or by Injecting* $10^4$ *W-256 Cells Intramuscularly* 2 *Weeks before IVI to Induce Solid Growth of Tumour in Leg of Each Rat (IMI). Six Rats in Each Group, Killed* 7 *days after IVI to Measure Tumour Growth*

| Group | Treatment LTI | IMI | HR | Mean final body weight (g) | Mean number of lung colonies (range) | Incidence of primary tumours (mean diameter) | Incidence of pelvic node metastases* |
|---|---|---|---|---|---|---|---|
| I | − | − | − | 152 | 5±2 (1–15) | — | — |
| II | − | + | − | 158 | 4±2 (0–15) | 6/6 (∼3 cm) | 0/6 |
| III | + | − | − | 154 | 191±45 (52–350) | — | — |
| IV† | + | + | − | 157 | 5±4 (0–24) | 6/6 (∼3·5 cm) | 2/6 |
| V | + | + | − | 146 | 1±0·6 (0–3) | 6/6 (∼3 cm) | 4/6 |
| VI | + | − | + | 151 | 2±1 (0–6) | — | — |

* Macroscopic presence of haemorrhagic tumour growth.
† No tumour cells were injected intravenously in Group IV.

general immunity with local reactions produced by the irradiation of lung tissue (the tumour bed). It is also considered significant that LTI (570–1750 rad) given immediately (<2 hours) before inoculation of weanlings caused slight but significant *decreases* in CFE. This effect was quite different from WBI which produced marked increases in CFE in weanlings (Fig. 2 and 6). These findings are considered to indicate also that LTI had no significant immunosuppressive action in affecting CFE. Its early effect is considered to consist of inhibition of growth of lung tissue—an effect which would be most marked in more rapidly

FIG. 8.—Tumour macrocolony counts in lungs and body weights of 8-week old female rats injected intravenously with 5 × $10^2$ W-256 cells 0–7 days after treatment with 0·5 ml ALS. Interrupted lines (C) indicate mean organ weights in untreated control rats (6 rats per point).

growing younger animals and which also produces temporarily less favourable conditions for take and growth of tumour cells. Indirect support for this hypothesis has been provided by measurements made of rates of DNA synthesis and cell proliferation in lungs after LTI, which have shown that these rates decrease at first but then increase to much higher than normal levels 7–10 days after 1000–1500 rad LTI (unpublished data), when the effect of LTI of increasing CFE is also most marked.

7. *ALS—inoculation interval*

The stimulating effect of a single intravenous dose of 0·5 ml heterologous rabbit-anti rat lymphocytic serum (ALS), on CFE in 8-week old rats given before inoculation decreased rapidly with increase in time between ALS and tumour cell injections and had practically disappeared in 7 days (Fig. 8). This rapid decrease in effect of ALS on CFE differed from that of LTI, which increased CFE as the time between LTI and inoculation was increased to a maximum at 7–14 days. ALS also differed from WBI and LTI in causing rapid, marked and sustained increases in specific weights of spleen, thymus, heart, lungs and kidneys. Also ALS increased the rates of [$^3$H]-thymidine,

FIG. 9.—Weights of primary tumour (Pr) and metastases in crural (CN), pelvic (PN) and upper abdominal (UAN) lymph nodes 7 days after intramuscular injection of $10^4$–$10^6$ Y-P388 or W-256 tumour cells in right leg of (a) unirradiated rats (closed symbols), (b) rats given 400 rad WBI 24 hours before transplantation (open symbols), (c) rats given 1000 rad to thorax 7 days before transplantation (LI, closed symbols) and (d) rats given both WBI and 1000 rad to thorax (LI; open symbols) (6 rats per point).

labelling of lung tissue DNA much more rapidly than LTI (to be published).

8. *Metastases in irradiated lungs*

W-256 or Y-P388 tumour cells were transplanted intramuscularly into the right calf muscle of 5-week old female rats. Growth of the primary tumour transplants and of metastases which developed in regional lymph nodes and lungs in unirradiated animals were compared with those in (i) rats with lungs given a single dose of 1000 rad 7 days preceding transplantation and (ii) rats given 570 rad WBI 24 hours preceding tumour transplantation (Fig. 9 and 10).

(a) *Y-P388.*—This tumour grows rapidly at the site of inoculation and metastasizes rapidly along lymphatic pathways to form solid haemorrhagic metastases in ipsilateral crural (CN), lower abdomino-pelvic (PN) and upper abdominal (UAN) lymph nodes. Cells which enter the thoracic duct are carried in the venous blood to the lungs, where the arrest and growth of these cells produces discrete macrocolonies within 7 days (van den Brenk *et al.*, 1971). The weights of individual lymph node metastases and number of lung colonies respectively are quantitatively related to the number of tumour cells transplanted and to the weight of the primary tumour.

Local irradiation of the lungs had no significant effect on rates of growth of primary tumour and lymph node metastases but caused significant increases in pulmonary metastases (Fig. 9 and 10). WBI increased growth of primary tumour

FIG. 10.—Same experiment as in Fig. 9 showing corresponding incidence of lung metastases, values for lung and spleen weights (same symbols used as in Fig. 9).

and metastases—particularly metastases in more distant UAN and lungs, where spread normally occurs later and consequently fewer cells deposit. Combined treatment with LTI and WBI enhanced the growth of lung metastases.

(b) *W-256.*—At the site of implantation in muscle this tumour grows as rapidly as Y-P388, but spread of the tumour to lymph nodes and lungs occurs much less frequently, even in immunosuppressed (WBI or ALS treated) rats, and only when a large number ($10^5$–$10^6$) of W-256 cells have been transplanted. LTI alone had no significant effect on growth of metastases in the lungs of this tumour, but combined with WBI produced marked increases in pulmonary metastases (Fig. 9 and 10).

9. *CFE for secondary challenge in irradiated lungs*

Five-week old rats were injected intramuscularly with $10^4$ W-256 cells to produce a solid tumour and stimulate immunity to tumour growth. On the same day as transplantation of the tumour was carried out, 1000 rad LTI was given. Seven days later $10^4$ W-256 cells were injected intravenously (secondary challenge) and each rat was killed 7 days after this injection in order to measure growth of primary tumour and metastases in lymph nodes, and to count the number of lung macrocolonies. These measurements were compared with those in groups of rats which had been inoculated intramuscularly or intravenously only, and with a group of rats given 3 intramuscular injections of $10^7$ HR (W-256) cells every second day for a week to stimulate immunity before intravenous injection with $10^4$ intact tumour cells. The results obtained (Table III) show that thoracic irradiation greatly increased the number of lung colonies (Group III) but not if the rats had been immunized by growth of the tumour in the leg or by HR tumour cells (Group V and VI), *i.e. local irradiation of the lungs did not interfere significantly with the expression of host immunity to proliferative growth of tumour cells in the lungs.*

### DISCUSSION

The results obtained have shown that under certain conditions local irradiation of pulmonary tissues greatly increases survival, take and clonogenic growth of tumour cells deposited by the blood in in the lungs—whether the cells are injected intravenously or disseminate to the lungs from growing solid tumour tissue present elsewhere in the animal. Stimulation of growth of tumour metastases by local lung irradiation is a strictly local phenomenon and is not due to the suppression of host immunity by the treatment. The action of LTI needs to be considered in conjunction with the finding that spontaneous resistance of host to the growth of a primary challenge with tumour cells in the lungs develops rapidly with increase in age after weaning, and when the rate of proliferative growth of normal tissues of the lungs decreases (van den Brenk *et al.*, 1973). A stimulating effect of local lung irradiation on CFE has been reported independently by others (Milas and Withers, 1970; Brown, 1971) using syngeneic systems in mice, in which immunological incompatibility between tumour and host was considered to be insignificant but CFE was low. Two hypotheses were advanced for the stimulating effect of LTI by these authors. Milas and Withers considered that tumour cells were trapped more readily in irradiated lungs and Brown attributed enhanced CFE " to an inactivation of the local macrophage scavenging system " in the lung by irradiation. Neither theory explains our results satisfactorily.

We have demonstrated that the immunological status of locally irradiated lung tissues remains relatively intact since LTI did not stimulate CFE in lungs of immunized rats. Furthermore, inactivation of cell-mediated immune reactions by irradiation should increase with increase in radiation dosage till the effect is maximal, but higher doses (<1500 rad) used by us reduced CFE (Fig. 5 and 7). Also, we have been unable to obtain histological evidence that lung macrophages decreased in number after LTI or that macrophage function was depressed when CFE had increased, namely 7 or more days after 1000 rad, or within 48 hours after WBI or ALS had been given (van den Brenk *et al.*, 1973). Indeed, 7–14 days after LTI, lungs in rats were found to capture more intravenously injected indian ink than unirradiated lungs (unpublished results) which could be taken to mean that local irradiation enhanced macrophage activities. The action of WBI is primarily immunosuppressive but WBI failed to increase CFE in older rats substantially, whereas LTI did not interfere with the expression of immunity, but stimulated CFE in the rat at all ages. We have shown previously that the proportion of viable W-256 tumour cells injected intravenously which do not trap in the lungs and escape into the systemic circulation is insignificant. Tumour colonies did not form in organs other than lungs, even under optimal conditions such as when immunity to tumour growth had been suppressed by WBI or ALS alone, or by both treatments combined, in weanling rats, treatments which raised CFE for this tumour to >0·1; nor were colonies seen to develop in organs other than the lungs after LTI. Preliminary measurements made, *in vitro* and *in vivo*, of capture and rates of loss from the lungs of intravenously injected W-256 cells labelled with DNA precursors, have shown also that immediate trapping and retention of the W-256 cells take place in both unirradiated and irradiated lungs. HR cells, added in excess to a viable W-256 cell inoculum did increase CFE, but the increase was relatively small (less than two-fold) compared with a 30–40 fold increase reported by Hill and Bush (1969) for a murine tumour. It has also been found that after LTI, addition of HR cells to a W-256 inoculum was even less effective in increasing CFE and it seemed unlikely that in the rat HR cells prevented fewer intact W-256 cells escaping from the lungs. Hill and Bush were able to increase CFE equally well by substituting inert plastic microspheres for HR cells. We have found that microspheres (10 $\mu$m or 25 $\mu$m diameter) added to W-256 cells were less efficient than HR cells in raising CFE (unpublished data).

We have shown that in order to stimulate CFE, LTI requires to be given several days before inoculation. A maximum stimulation develops 7–14 days after irradiation, and thereafter decreases rapidly. At 7–14 days after LTI, radiation reactions associated with inflammatory changes have developed in the lungs and overlying tissues. When these reactions decreased subsequently and gave way to reparative fibrosis, CFE also decreased. It is postulated that biochemical changes associated with cell proliferative activity of normal tissues play an essential part in the "take" and growth of single tumour cells (metastases) in the lungs and that this factor is the principal mechanism whereby local irradiation stimulates CFE. The degree of stimulation of CFE after local irradiation was closely related to the increase in rate of DNA synthesis in the lungs which developed after LTI (to be published). Attenuation and delay of the acute inflammatory reactions to radiation of the lungs by treatment with anti-inflammatory steroids, have been found to reduce CFE (unpublished results). Previous results obtained have suggested that stimulation of CFE of tumour in lungs by immunosuppressive agents (WBI and ALS) may also be partly due to local reactive changes in the damage these agents cause in the normal tissues of the lung and we have postulated that growth stimulating substances (GSS) increase in concentration as a result of such reactions, and can act locally *in vivo*. Also, GSS are considered to be present in higher concentrations in the more rapidly growing organs of younger growing animals. Their growth promoting action in raising CFE is considered to be similar in nature to that of "feeder" cells *in vitro* and the high CFE obtained in lungs of weanling rats also is considered to depend largely on increased concentrations of GSS present in less mature, more rapidly growing tissues (van den Brenk and Sharpington, 1972; van den Brenk *et al.*, 1973).

It seems highly probable that a high proportion of tumour cells which enter the blood stream of humans and other mammals with neoplastic disease may fail to produce metastases. This may be due to surveillance mechanisms which actively destroy tumour cells (*e.g.* immunity) but may also be due to an inability of cells to "thrive" in foreign tissues, where the biochemical milieu fails to provide the factors essential for their establishment and growth. In younger animals, tumour transplants often grow better and tend to spread more rapidly and produce overt metastases more readily. Incompletely developed immunological functions in younger animals may contribute to this situation, particularly if tumour and host are immunologically incompatible, but other physiological factors also seem to be involved and may play a significant role in the "destruction" of circulating tumour cells.

The fact that irradiated lung tissues are better able to support growth of single cancer cells raises the important question of the possible enhancement of pulmonary metastases (and indeed metastases in other organs) by local irradiation in the human. Attention has been drawn to increased frequency of metastases in patients receiving radiotherapy for breast or lung cancer (Paterson and Russell, 1959, 1962). Other surveys (Bond, 1967; Fisher *et al.*, 1971) have suggested that post-operative radiotherapy for breast cancer increases the incidence and rates of development of metastases in neighbouring organs. The mechanism involved has most frequently been considered to be suppression of tumour autoimmunity by irradiation. However, further studies seem warranted, based on the notion that nutritional requirements for clonogenic growth of cells of different tumours may vary, and that the physiological and pathological condition of a target tissue may determine its capacity to meet these requirements.

### REFERENCES

BOND, W. H. (1967) The Influence of Various Treatments on Survival Rates in Cancer of the Breast. In *The Treatment of Carcinoma of the Breast*. Ed. A. S. Jarrett. London: Excerpta Medica. p. 24.

BROWN, J. (1971) Effects of Lung irradiation on the Incidence of Lung Colonies Arising from Intravenously Injected KHT Tumour Cells (abstract). *Radiat. Res.*. **47**, 271.

ELKIND, M. M. & WHITMORE, G. F. (1967) *The Radiobiology of Cultured Mammalian Cells.* New York: Gordon and Breach.

FISHER, B., SLACK, N. H., CAVANAUGH, P. J., GARDNER, B. & RAVDIN, R. G. (1971). Postoperative Radiotherapy in the Treatment of Breast Cancer: Results of the NSABP Clinical Trial. *Ann. Surg.*, **172**, 711.

HILL, R. P. & BUSH, R. S. (1969) A Lung Colony Assay to Determine the Radiosensitivity of the Cells of a Solid Tumour. *Int. J. Radiat. Biol.*, **15**, 435.

MILAS, A. & WITHERS, R. (1970) Abstract 566. Report Fourth International Congress of Radiation Research, Evian.

PATERSON, R. & RUSSELL, M. H. (1959) Clinical Trials in Malignant Disease, pt. II, III. Breast Cancer. *J. Fac. Radiol.*, **10**, 130, 175.

PATERSON, R. & RUSSELL, M. H. (1962) Clinical Trials in Malignant Disease. IV. Lung Cancer. *Clin. Radiol.*, **13**, 141.

VAN DEN BRENK, H. A. S. & MOORE, V. (1971) Lung Colony Assays of Tumour Cells in Immunologically Intact and Suppressed Rats. *Int. J. Radiat. Biol.*, **20**, 185.

VAN DEN BRENK, H. A. S., MOORE, V. & SHARPINGTON, C. (1971) Growth of Metastases from P-388 Sarcoma in the Rat Following Whole Body Irradiation. *Br. J. Cancer*, **25**, 186.

VAN DEN BRENK, H. A. S. & SHARPINGTON, C. (1972) Effect of Local X-irradiation of a Primary Sarcoma in the Rat on Dissemination and Growth of Metastases: Dose-Response Characteristics. *Br. J. Cancer*, **25**, 812.

VAN DEN BRENK, H. A. S., SHARPINGTON, C. & ORTON, C. (1973) Macrocolony Assays in the Rat of Allogeneic Y-P388 and W-256 Tumour Cells Injected Intravenously: Dependence of Colony Forming Efficiency on Age of Host and Immunity. *Br. J. Cancer*, **27**, 134.